# High-Throughput Genetic and Gene Expression Analysis of the RNAPII-CTD Reveals Unexpected Connections to SRB10/CDK8

**DOI:** 10.1371/journal.pgen.1003758

**Published:** 2013-08-29

**Authors:** Maria J. Aristizabal, Gian Luca Negri, Joris J. Benschop, Frank C. P. Holstege, Nevan J. Krogan, Michael S. Kobor

**Affiliations:** 1 Centre for Molecular Medicine and Therapeutics, Child and Family Research Institute, Department of Medical Genetics, University of British Columbia, Vancouver, British Columbia, Canada; 2 School of Medicine and Medical Science, University College Dublin, Belfield, Dublin, Ireland; 3 Molecular Cancer Research, University Medical Centre Utrecht, Utrecht, The Netherlands; 4 Department of Cellular and Molecular Pharmacology, University of California, San Francisco, San Francisco, California, United States of America; Harvard Medical School, United States of America

## Abstract

The C-terminal domain (CTD) of RNA polymerase II (RNAPII) is composed of heptapeptide repeats, which play a key regulatory role in gene expression. Using genetic interaction, chromatin immunoprecipitation followed by microarrays (ChIP-on-chip) and mRNA expression analysis, we found that truncating the CTD resulted in distinct changes to cellular function. Truncating the CTD altered RNAPII occupancy, leading to not only decreases, but also increases in mRNA levels. The latter were largely mediated by promoter elements and in part were linked to the transcription factor Rpn4. The mediator subunit Cdk8 was enriched at promoters of these genes, and its removal not only restored normal mRNA and RNAPII occupancy levels, but also reduced the abnormally high cellular amounts of Rpn4. This suggested a positive role of Cdk8 in relationship to RNAPII, which contrasted with the observed negative role at the activated *INO1* gene. Here, loss of *CDK8* suppressed the reduced mRNA expression and RNAPII occupancy levels of CTD truncation mutants.

## Author Summary

RNA Polymerase II (RNAPII) is the enzyme responsible for the transcription of all protein-coding genes. It has a unique extended domain called the C-terminal domain (CTD). This domain is highly conserved across species and is composed of repeats of a seven amino acid sequence. The CTD functions as a recruiting platform for regulatory and RNA processing factors, making the CTD a master orchestrator of transcription. Previous work revealed a critical role for CTD length in the transcription of induced genes. However, how CTD length is generally required for transcription is currently unclear, as is the mechanism underlying the observed suppression of CTD truncation phenotypes by loss of the *SRB10/CDK8* gene. Here, using gene expression microarrays, we determined the set of genes most sensitive to alternations in CTD function and uncovered unexpected links between RNAPII-CTD and Cdk8.

## Introduction

The largest subunit of RNA polymerase II, Rpb1, has a unique C-terminal domain (CTD) composed of the repeated sequence Tyr-Ser-Pro-Thr-Ser-Pro-Ser (Y_1_ S_2_ P_3_ T_4_ S_5_ P_6_ S_7_) [1], [2]. Although the CTD is highly conserved across species, the number of repeats varies in a manner resembling genomic complexity, with 25/26 repeats in *Saccharomyces cerevisiae* and 52 in humans [3]. Deletion of the entire CTD is lethal in budding yeast, while strains carrying 9–13 repeats are viable but display conditional phenotypes [4], [5]. While not required to support basal transcription *in vitro*, the CTD is critical for the response to activator signals *in vivo* [6], [7]. For example, CTD truncation mutants exhibit reduced activation of *INO1* and *GAL10* upon switching to inducing conditions [7].

The CTD is a scaffold for the recruitment of RNA processing and chromatin remodeling factors, a function linked to its differential phosphorylation at specific residues of the heptapeptide repeat [3]. Transcription begins with the recruitment of RNAPII with an unphosphorylated CTD to promoters, where it interacts with components of the transcription pre-initiation complex (PIC) [8], [9]. Following, it is phosphorylated at S_5_ and S_7_ by the general transcription factor TFIIH, facilitating recruitment of capping enzymes and release of RNAPII from promoter-bound elements [10]–[13]. Elongation is characterized by phosphorylation of S_2_ by Ctk1 and Y_1_ and T_4_ by yet unidentified kinases [14], [15]. S_2_ and Y_1_ phosphorylation play a role in the temporal recruitment of elongation and termination factors [15]. Subsequently, termination entails removal of all phosphorylation marks by Fcp1 and Ssu72 to regenerate an initiation competent RNAPII molecule [16]–[18].

While early work aimed at understanding CTD function uncovered a set of *SRB* (Suppressor of RNA Polymerase B) genes, a comprehensive genetic network governing CTD function has yet to be fully elucidated [19]. Of the identified *SRB* genes many encode members of a large multisubunit complex known as Mediator [20]. Mediator was first identified *in vitro* as a cellular fraction that stimulates RNAPII transcription, and is now known to not only physically interact with the CTD, but also to be important for the response to up-stream regulatory signals [21]. Although primarily associated at RNAPII gene promoters, Mediator also resides at open reading frames (ORFs) [22], [23]. Furthermore, Mediator is organized into four functionally distinct submodules: head, middle, tail and Cdk8 module [24]. The head module interacts with the CTD while the tail and middle modules interact with gene-specific and general transcription factors [25], [26]. The Cdk8 kinase module likely associates transiently with the core Mediator complex and has roles in both transcriptional activation and repression [27], [28]. This dual activity is in part mediated by Cdk8's ability to phosphorylate multiple regulatory components of the transcription machinery. These include several transcription factors as well as factors more generally required for transcription such as the CTD itself [27], [29]–[31]. While the mechanistic role of some of these phosphorylation events is unclear, CTD phosphorylation by Cdk8 prior to promoter association inhibits RNAPII recruitment and transcription initiation *in vitro* [29]. In contrast, CTD phosphorylation by Cdk8 and Kin28 following promoter association promotes RNAPII release from the PIC and thus stimulates transcription activation [30].

The work here highlighted the functional circuitry between the RNAPII-CTD and Mediator in the regulation of cellular homeostasis, gene expression, and the transcription factor Rpn4. Our data uncovered a length-dependent requirement of the CTD for genetic interactions and mRNA levels of genes expressed under normal growth conditions. Truncating the CTD primarily resulted in increased expression and RNAPII association at a subset of genes, in part mediated by changes to transcription initiation. These genes had preferential association of Cdk8 at their promoters and were regulated by the transcription factor Rpn4. The expression and RNAPII binding defects of the majority of this subset of genes were suppressed by deleting *SRB10/CDK8*, suggesting that in CTD truncation mutants, Cdk8 functioned to enhance transcription and RNAPII association at a subset of genes. Conversely, our data also revealed that deletion of *CDK8* suppressed the activation defects of CTD truncation mutants at the *INO1* locus thus indicating that Cdk8 also functioned to repress transcription and RNAPII association in CTD truncation mutants.

## Results

### The RNAPII CTD Was Linked to an Extensive Genetic Interaction Network

**Figure 1:**
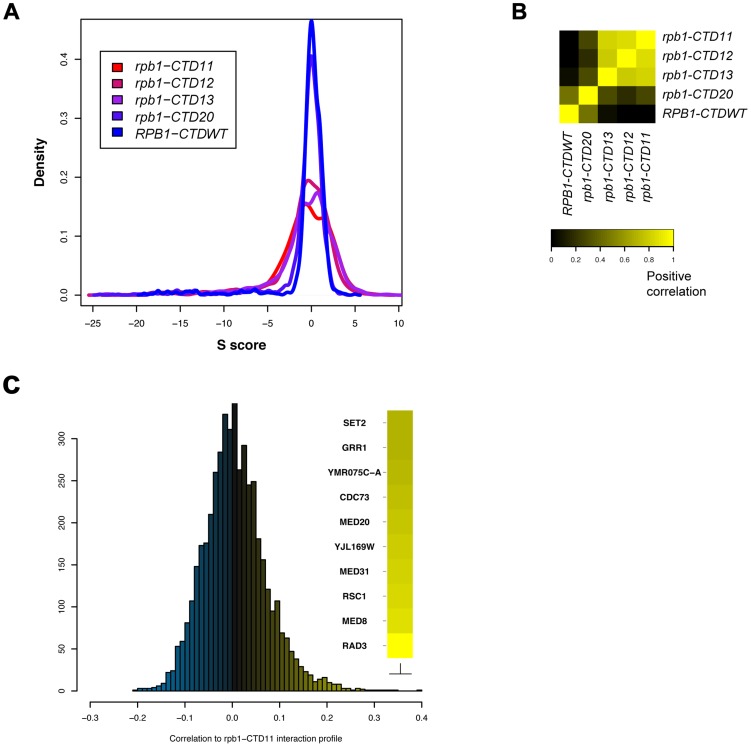
E-MAP uncovered CTD length-dependent genetic interactions with genes involved in transcription. The genetic interaction profile of strains containing 11, 12, 13 or 20 heptapeptide repeats (*rpb1-CTD11*, *rpb1-CTD12*, *rpb1-CTD13* and *rpb1-CTD20*) against a library of 1532 different mutants involved principally in aspects of chromatin biology and RNA processing. CTD truncations were created at the endogenous *RPB1* locus by addition of a TAG stop codon followed by a NAT resistance marker. *RPB1-CTDWT* served as a control and contained a NAT resistance marker following the endogenous stop codon (A) Distribution of S scores for CTD truncation mutants revealed an increase in the number of significant genetic interactions as a result of truncating the CTD. The S score is a modified T-statistic measure, which captures both the confidence and strength of the genetic interaction. Scores greater than 2.0 or less than −2.5 are considered significant. (B) Spearman rho correlation of CTD truncated mutants identified two distinct groups (C) Distribution of Pearson's correlation scores derived from comparing the *rpb1-CTD11* interaction profile to all previously assayed strains.

To broadly determine the requirement of CTD length for cellular function, we used Epistasis Mini Array Profiling (E-MAP) to generate genetic interaction profiles of CTD truncation mutants containing 11, 12, 13 or 20 heptapeptide repeats (*rpb1-CTD11*, *rpb1-CTD12*, *rpb1-CTD13* and *rpb1-CTD20* respectively) against a library of 1532 different mutants involved principally in aspects of chromatin biology and RNA processing [32] (Table S1). CTD truncations were created at the *RPB1* locus by addition of a TAG stop codon followed by a NAT resistance marker. As a control for the genetic integration strategy we also generated *RPB1-CTDWT*, which contained a NAT resistance marker following the endogenous stop codon. While the minimal CTD length for viability is 8 repeats, we focused on strains starting at 11 repeats as mutants bearing shorter CTDs were significantly unstable in our hands, consistent with previous findings [33]. Overall our data revealed a greater number of significant genetic interactions as the CTD was progressively shortened, an effect consistent with increasingly disrupted function (Figure 1A). Furthermore, while hierarchical clustering based on Spearman's rho correlation delineated two major clusters, the first including *rpb1-CTD11*, *rpb1-CTD12* and *rpb1-CTD13* and the second consisting of *rpb1-CTD20* and *RPB1-CTDWT* (Figure 1B), individual genetic interactions revealed more nuanced CTD length-dependent genetic interaction patterns (Figure S1). For example, aggravating interactions were observed with strains lacking *ASF1*, *RTT109* and *DST1* when the CTD was truncated to 13 repeats or shorter, while truncation to 11 repeats was required for aggravating interactions with *SET2*, *RTR1* and *SUB1*. Collectively, this data revealed significant and specific functional alterations to the CTD as a result of shortening its length and suggested that individual pathways required different CTD lengths for normal function. Finally, given that we identified significant genetic interactions with genes involved in a variety of processes, we compared the E-MAP profile of our shortest CTD truncation with all previously generated profiles to determine which pathways were principally affected by truncating the CTD. This analysis revealed that four of the ten most correlated profiles belonged to loss of function alleles of genes encoding subunits of TFIIH and Mediator (*RAD3*, *MED8*, *MED31* and *MED20*) suggesting that shortening the CTD results in genetic interaction patterns most similar to mutants affecting transcription initiation (Figure 1C).

### CTD Serial Truncations Led to Progressive Changes in Transcription

**Figure 2:**
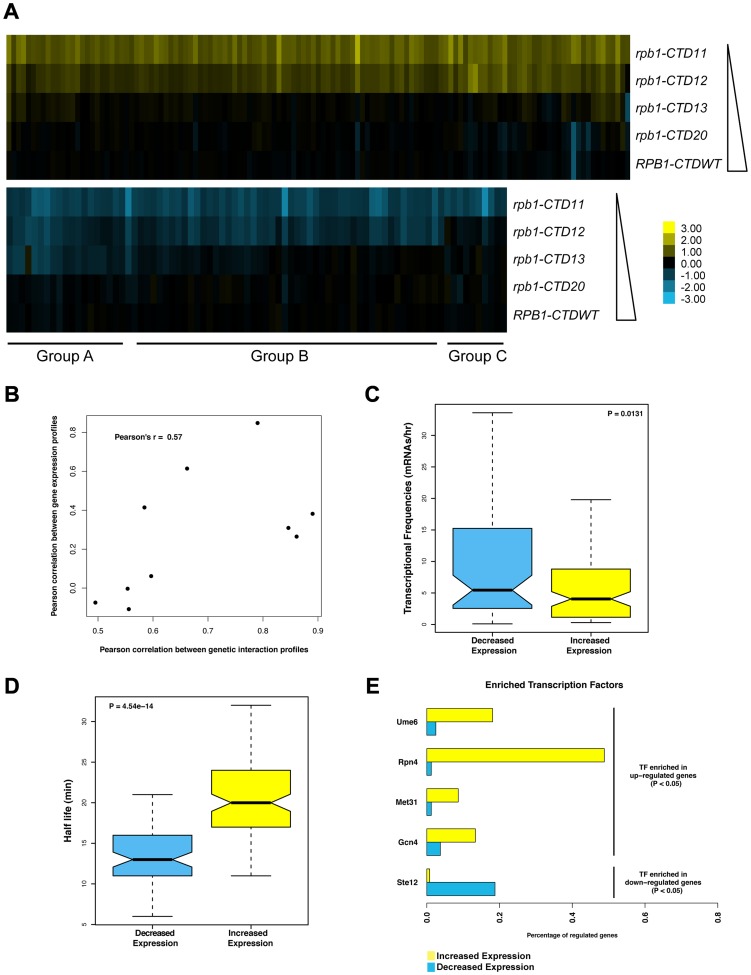
Serial CTD truncations led to progressive steady state transcriptional defects. Expression microarrays were normalized using spiked in controls to determine global changes in mRNA levels. As no such changes were detected, the expression profiles were normalized to total mRNA levels. Differentially expressed genes were determined by p value <0.01 and fold change >1.7 compared to wild type. (A) Heatmap of genes with significantly increased (top) or decreased (bottom) mRNA levels in the *rpb1-CTD11* mutant. Groups A, B and C approximately outline subsets of genes whose expression were decreased when the CTD was truncated to 13, 12 or 11 repeats respectively. Yellow indicates genes with increased mRNA levels and blue indicates genes with decreased levels. (B) Scatterplot of profile paired correlations in gene expression and genetic interaction. Boxplot of transcriptional frequency (C) and mRNA half-life (D) showing significant differences in half-life (p value 4.54e-14) and transcriptional frequency (p value 0.0131) between genes with increased or decreased expression in the *rpb1-CTD11* mutant. Outliers are not shown. (E) Differences in enriched transcription factors between genes with increased or decreased mRNA levels.

Although the CTD plays a major role in the response to activator signals *in vivo*, its general involvement in transcription is less well defined. To investigate this important aspect, we generated gene expression profiles of CTD truncation mutants in normal growth conditions (Table S2) (Complete dataset can be found in array-express, code E-MTAB-1431). Similar to the E-MAP data, the expression data revealed a length-dependent requirement for CTD function, with the severity and number of transcriptional changes increasing as the CTD was progressively shortened (comparison of E-MAP vs. expression profiles Pearson's rho 0.57) (Figure 2A and 2B). This gradient effect was clearly visible in the group of genes whose transcript levels decreased upon truncation of the CTD (Figure 2A groups A, B and C constitute genes requiring greater than 13, 12, and 11 repeats for normal transcription respectively), and thus provided strong evidence of a gene-specific CTD length requirement for normal transcription. Surprisingly, given the central role of the CTD in RNAPII function, our microarray data identified only 127 genes with significant increases in mRNA levels and 80 genes with significant decreases (p value <0.01 and fold change >1.7 compared to wild type), in strains carrying the shortest CTD allele, *rpb1-CTD11*. Functional characterization of the set of genes with increased and decreased mRNA levels suggested that the transcriptional alterations were not affecting a random group of genes. Instead, using previously published transcription frequency data, we found that the genes with decreased mRNA levels tended to be highly transcribed with short mRNA half-lives, while the genes with increased mRNA levels were mostly lowly transcribed with long mRNA half-lives (Figure 2C and 2D) [34]. In addition, these genes belonged to different functional gene ontology (GO) categories. The genes with increased mRNA levels were enriched for proteasome and proteasome-associated catabolism processes while the genes with decreased levels were enriched for iron homeostasis, purine metabolism and pheromone response (Table S3). Finally, these genes were differentially regulated by transcription factors (Figure 2E). The genes whose expression levels decreased were principally bound by Ste12, while those with increased expression were bound by Ume6, Met31, Gcn4 and most significantly by Rpn4 which bound 46% of these genes (p value 1.46E-41).

### Truncating the RNAPII CTD Had Varying Effects on the Genome-Wide Occupancy Profile of Transcription Related Factors

**Figure 3:**
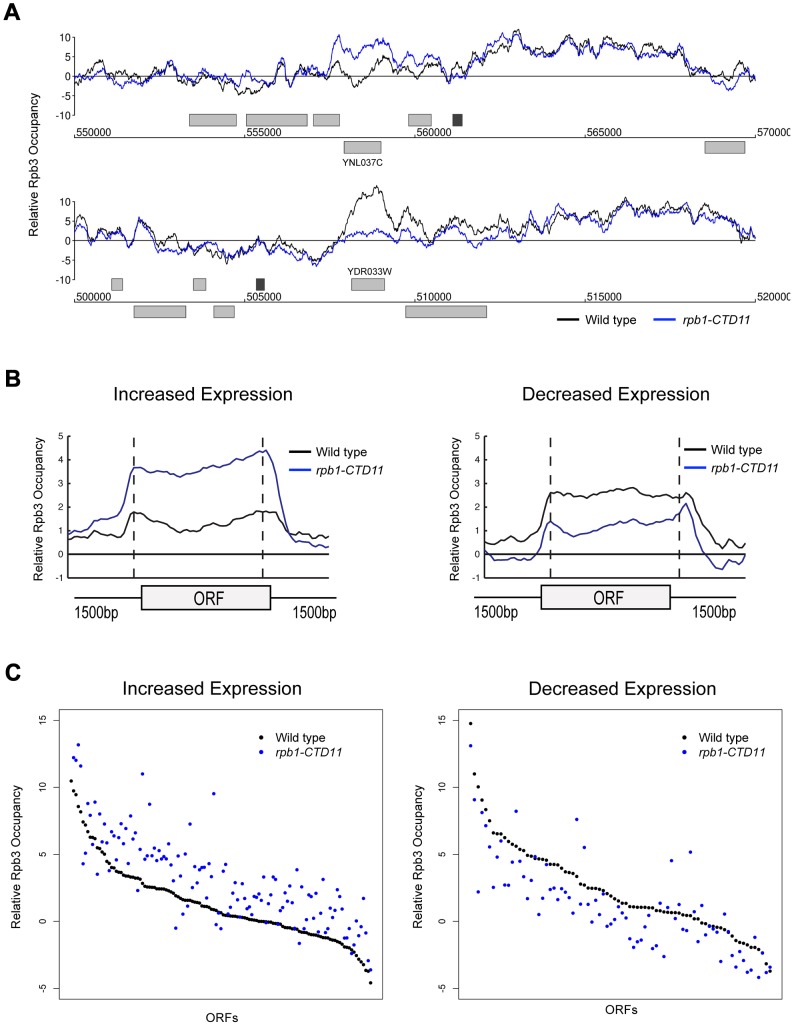
Genome-wide occupancy profiles of RNAPII identified a direct effect for the CTD in transcription regulation. (A) Chromosome plots of relative Rpb3 occupancy revealed similar profiles between wild type and *rpb1-CTD11* mutants. Rpb3 occupancy differences were observed in the *rpb1-CTD11* mutant at genes identified to have significantly increased (YNL037C - top) or decreased (YDR033W - bottom) mRNA levels. Light gray boxes depict ORFs and dark gray boxes depict ARSs. (B) Average gene profile of Rpb3 in genes with increased (left) or decreased (right) mRNA levels upon truncation of the CTD. (C) Average Rpb3 occupancy scores at coding regions with increased (left) (p value 3.36e-7) or decreased (right) (p value 2.98e-22) mRNA levels revealed an intimate link between Rpb3 binding and expression levels.

The measured gene expression changes in CTD truncation mutants could result from either effects on the synthesis or stability of the mRNA. To differentiate between these two possibilities, we measured RNAPII occupancy genome-wide and determined if the changes in gene expression correlated with alterations in RNAPII occupancy (Complete dataset can be found in array-express, code E-MTAB-1341). Specifically, we measured RNAPII in *rpb1-CTD11* and wild type cells by chromatin immunoprecipitation followed by hybridization on a whole genome tiled microarray (ChIP-on-chip) using an antibody specific to the RNAPII subunit Rpb3. Despite the use of different platforms, antibodies and normalization methods, the obtained genome-wide Rpb3 occupancy profiles obtained in wild type cells were highly correlated with those previously published by several groups (Figure S2) [35]–[39]. Furthermore, the occupancy maps revealed highly correlated profiles between *rpb1-CTD11* and wild type cells (Spearman's rho 0.85), agreeing with the limited transcriptional differences detected by the expression analysis. Nonetheless, our Rpb3 occupancy plots showed clear RNAPII occupancy differences along genes that were identified as either having increased or decreased mRNA levels in the *rpb1-CTD11* mutant (Figure 3A and B). Accordingly, plotting the average Rpb3 occupancy scores of the differentially regulated genes in *rpb1-CTD11* versus wild type cells revealed that the genes with increased mRNA levels had a significant increase in Rpb3 binding levels along their coding regions while the genes with decreased mRNA levels had a significant decrease (one-tailed t-test p value 2.98e-22 and 3.36e-7, respectively), thus suggesting a direct effect of truncating the CTD on RNAPII levels and mRNA synthesis at specific loci (Figure 3C).

**Figure 4:**
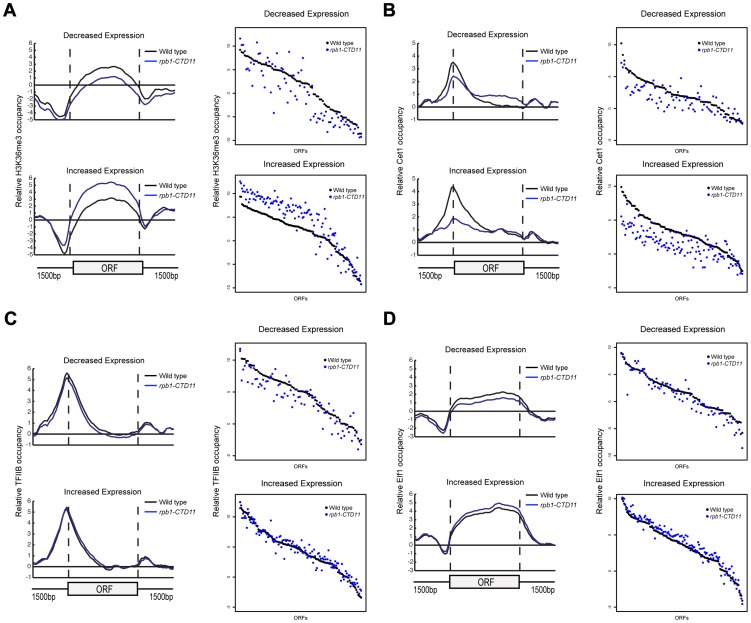
The RNAPII CTD was critical for the association of transcription related factors. (A, B, C and D) Left. Average gene profiles of H3K36me3, Cet1, TFIIB and Elf1 at genes with decreased (top) or increased (bottom) mRNA levels upon truncation of the CTD. Right. Average occupancy scores of H3K36me3, Cet1, TFIIB and Elf1 at genes with decreased (top) (paired t-test p value 8.68e-6, 2.72e-7, 8.66e-8 and 9.17e-6 respectively) or increased (bottom) (paired t-test p value 9.34e-23, 7.82e-25, 0.136 and 4e-15 respectively) mRNA levels upon truncation of the CTD. For H3K36me3 and Efl1, the average occupancy scores were calculated for the coding region. For Cet1 and TFIIB, the average occupancy scores were calculated for the promoter, which consisted of 500 bp upstream of the start codon.

To better understand the effect of truncating the CTD on transcription, we generated genome-wide association profiles of representative transcription associated factors. These factors included the initiation factor, TFIIB which is encoded by the *SUA7* gene, the capping enzyme Cet1, the elongation factor Elf1, and the Set2-dependent elongation associated chromatin mark histone H3 lysine 36 trimethylation (H3K36me3) (Complete dataset can be found in array-express, code E-MTAB-1379). We note that with the exception of *CET1* (which was not present on our E-MAP array), the genes encoding these factors had negative genetic interactions with our shortest CTD truncation allele. Our genome-wide occupancy profiles under wild type conditions were highly correlated to those previously reported (Figure 4 and Figure S3) [35], [40]. Overall, genome-wide occupancy was independent of CTD length for TFIIB, Elf1 and H3K36me3, despite the latter having decreased bulk levels in CTD truncation mutants (Figure S3) [41]. In contrast, Cet1 chromatin association decreased primarily in genes with lower transcriptional frequencies, perhaps reflective of its decreased binding to RNAPII with a shortened CTD (Figure S3B) [42]. Focusing on only the genes whose expression levels were altered in the CTD truncation mutants, we observed several interesting patterns. First, the levels of H3K36me3 correlated well with the transcription changes as its occupancy was decreased in genes whose expression decreased and increased in genes whose expression increased in the *rpb1-CTD11* mutant (paired t-test p value 8.68e-6 and 9.34e-23 respectively) (Figure 4A). Second, the levels of Cet1 were greatly reduced at the promoters of genes whose expression increased in *rpb1-CTD11* while only slightly reduced at those whose expression decreased (Figure 4B) (paired t-test p value 7.82e-25 and 2.72e-7 respectively). Lastly, both TFIIB and Elf1 had statistically significant CTD-length dependent occupancy changes, although the overall magnitude of change was minor compared to that of H3K36me3 and Cet1 (Figure 4C and D).

### Increases in mRNA Levels in CTD Truncation Mutants Were in part a Result of Increased Transcription Initiation

**Figure 5:**
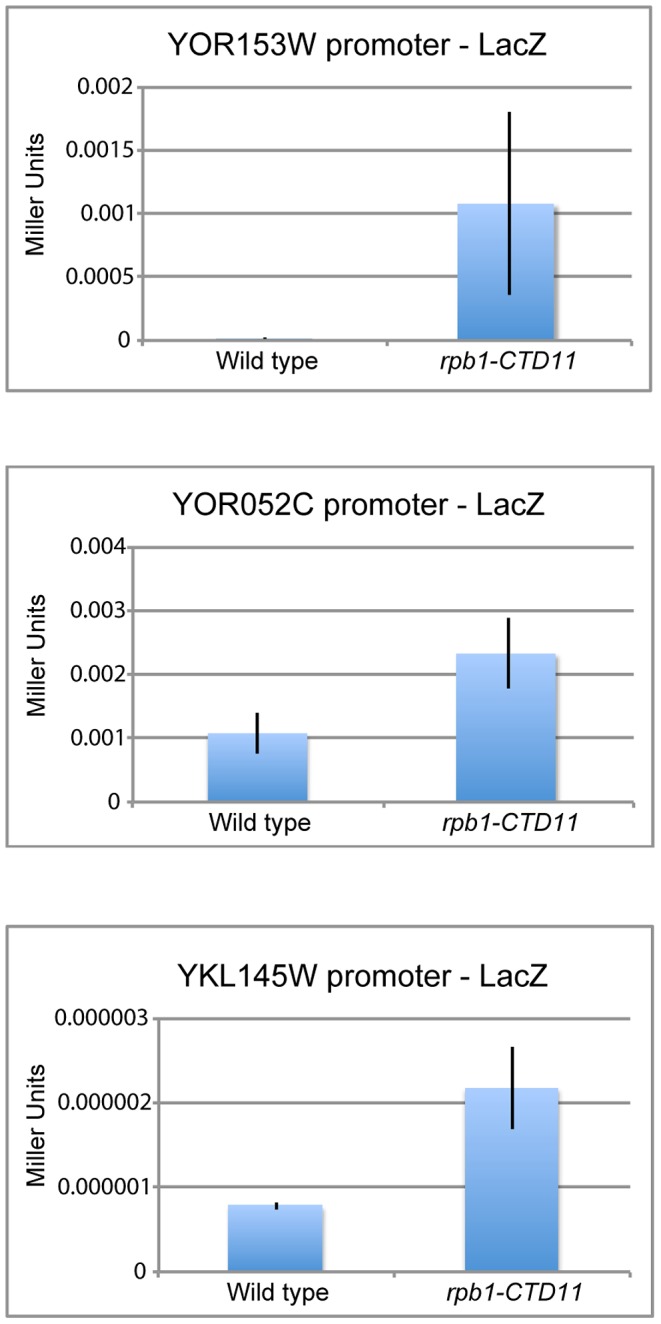
Increases in mRNA levels in CTD truncation mutants were in part a result of increased transcription initiation. Reporter assays showed that 450 bp of promoter sequence were sufficient to recapitulate the expression levels of three genes with increased mRNA levels in the *rpb1-CTD11* mutant.

The genetic similarity of CTD truncation mutants with mutants encoding initiation factors along with the ChIP-on-chip profiles of RNAPII and transcription associated factors suggested that possible changes to transcription initiation in the CTD truncation mutants might mediate some of the effects on gene expression. Using a LacZ reporter gene strategy we tested if the promoter elements of a set of exemplary genes sufficed to recapitulate the observed changes in expression. These assays revealed significant increases in β-galactosidase activity when the promoter regions of a subset of genes with increased mRNA levels were tested in the *rpb1-CTD11* mutant compared to wild type. These data confirmed that alterations to promoter-directed initiation events were in part responsible for the increased expression observed for these genes at their native loci (Figure 5). In contrast, the promoters of the genes with decreased mRNA levels in *rpb1-CTD11* mutants showed no significant differences in β-galactosidase as compared to wild type cells.

### Deletion of *CDK8* Normalized mRNA and RNAPII Levels at a Subset of *Rpb1-CTD11* Mis-regulated Genes

**Figure 6:**
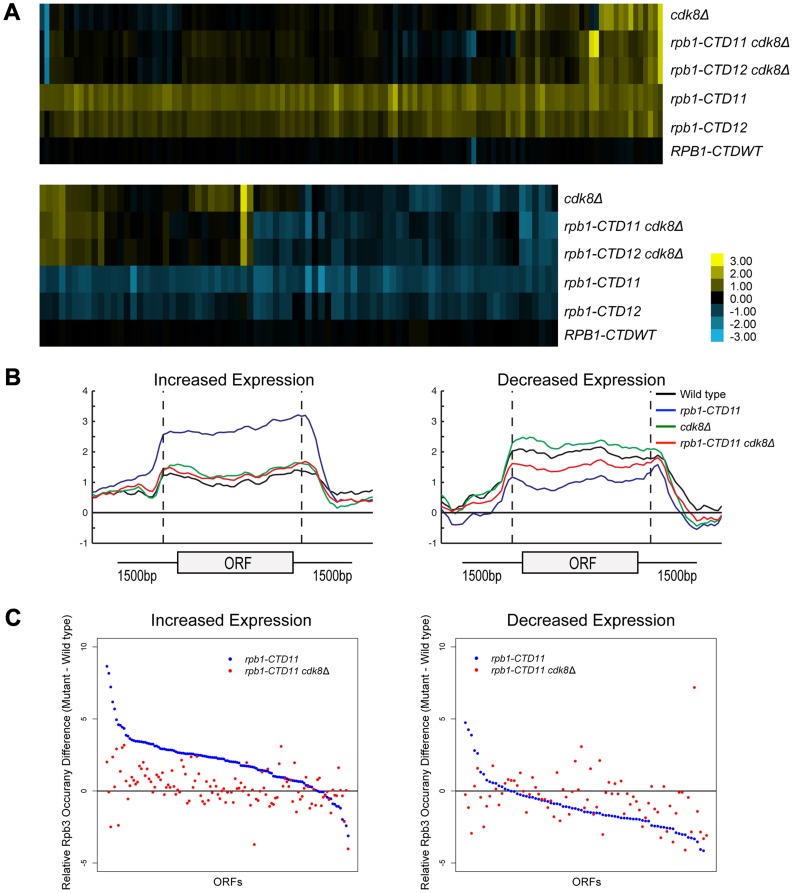
Loss of *CDK8* normalized *rbp1-CTD11* transcriptional defects by altering RNAPII recruitment.

We next expanded our characterization of the CTD to explore the well-established connection to Cdk8 in more detail. First, we showed that in addition to suppressing the cold sensitive phenotype of CTD truncation mutants, loss of *CDK8* could also suppress other known CTD growth defects (Figure S4) [19]. Second, despite Cdk8 being able to phosphorylate the CTD, its loss had only very minor effects on the bulk CTD phosphorylation defects seen in CTD truncation mutants [43], [44] (Figure S4). Third, we found that loss of *CDK8* had striking effects on the mRNA levels of genes whose expression was dependent on the CTD. Specifically, comparison of mRNA expression profiles for *rpb1-CTD11 cdk8Δ* and *rpb1-CTD12 cdk8Δ* double mutants to the single mutants revealed wide-spread and robust restoration of most of the genes with increased mRNA levels in *rpb1-CTD11*, while only a few of the genes with decreased mRNA levels appeared to be suppressed (Figure 6A). The restoration of mRNA levels in the genes with increased expression in the *rpb1-CTD11* mutant was mediated by regulation of RNAPII levels, as Rpb3 occupancy changed from an elevated state in the *rpb1-CTD11* mutant to close to wild type levels in the *rpb1-CTD11 cdk8Δ* mutant (Figure 6B). Accordingly, the average Rpb3 binding scores at these genes in the *rpb1-CTD11 cdk8Δ* mutant were significantly lower than the scores of the *rpb1-CTD11* mutant and were not statistically different from the scores of wild type cells (one-tailed t-test p value 7.17e-18 and 0.159 respectively) (Figure 6C). Consistent with fewer genes being suppressed in the set of genes with decreased mRNA levels in the *rpb1-CTD11* mutant, a restoring effect on RNAPII levels was not observed at this set of genes (Figure 6C).

**Figure 7:**
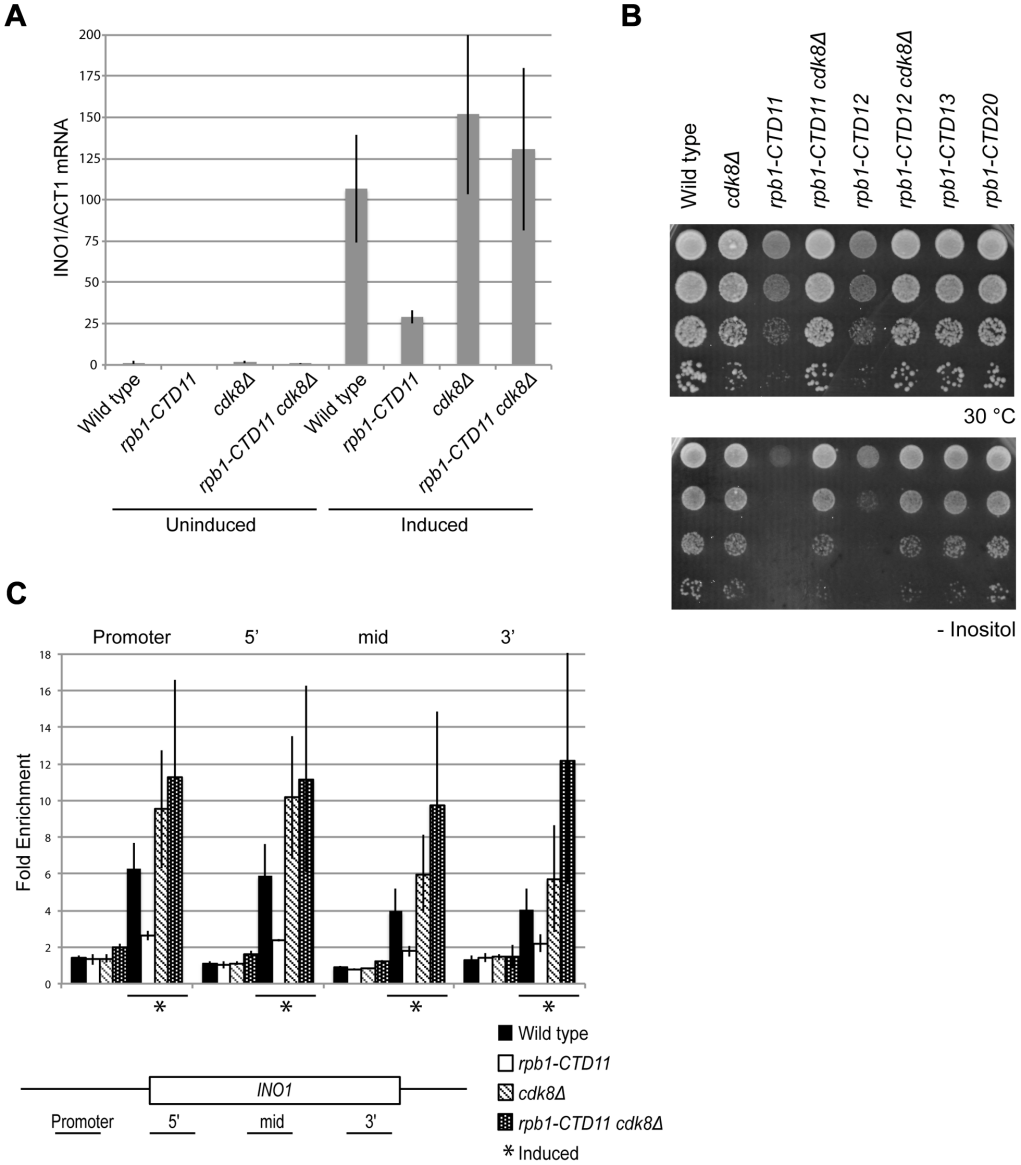
*INO1* expression and RNAPII association defects of *rpb1-CTD11* mutants were suppressed by deleting *CDK8*. Cells were grown in inositol containing media (200 µM) to constitute the uninduced sample, and shifted to inositol deplete media for 4 hrs to constitute the induced sample. (A) qRT-PCR analysis of *INO1* expression revealed a restoration of expression upon loss of *CDK8*. *INO1* mRNA levels were normalized to *ACT1* levels. (B) The sensitivity of CTD truncation mutants containing 11 or 12 repeats to growth in media lacking inositol was suppressed by deleting *CDK8*. (C) ChIP analysis of Rpb3 binding along the *INO1* gene. Asterisks indicate induced conditions. Rpb3 enrichment along the *INO1* gene was normalized to an intergenic region of chromosome V. Error bars represent standard deviations of values from three replicates.

A previously characterized phenotype of CTD truncation mutants is reduced activation of *INO1* and *GAL10* upon switching to inducing conditions. Therefore, we investigated if loss of *CDK8* could also suppress these expression defects of CTD truncation mutants [7]. Focusing on *INO1*, a gene important for the synthesis of inositol and survival in response to inositol starvation, we measured *INO1* mRNA levels in wild type, *rpb1-CTD11*, *cdk8Δ* and *rpb1-CTD11 cdk8Δ* mutants before and after induction. In agreement with previous work, *rpb1-CTD11* mutants had an impaired ability to activate *INO1* expression upon induction (Figure 7A) [7], [45]. Upon deletion of *CDK8*, *INO1* mRNA levels were robustly and reproducibly restored. This effect was corroborated with the suppression of the growth defect of CTD truncation mutants in media lacking inositol upon removal of *CDK8* (Figure 7B). Consistent with this being a direct effect on mRNA synthesis, Rpb3 levels throughout the *INO1* gene in *rpb1-CTD11* mutants were significantly lower as compared to wild type. Furthermore, upon deletion of *CDK8*, the levels of RNAPII associated with the *INO1* gene were restored (Figure 7C). While not statistically significant, we nevertheless observed a tendency for increased Rpb3 occupancy at the 3′ end of the gene in *cdk8Δ* and *rpb1-CTD11 cdk8Δ* mutants.

### Genes with Increased mRNA Levels in the *rpb1-CTD11* Mutant Were Directly Regulated by Cdk8

**Figure 8:**
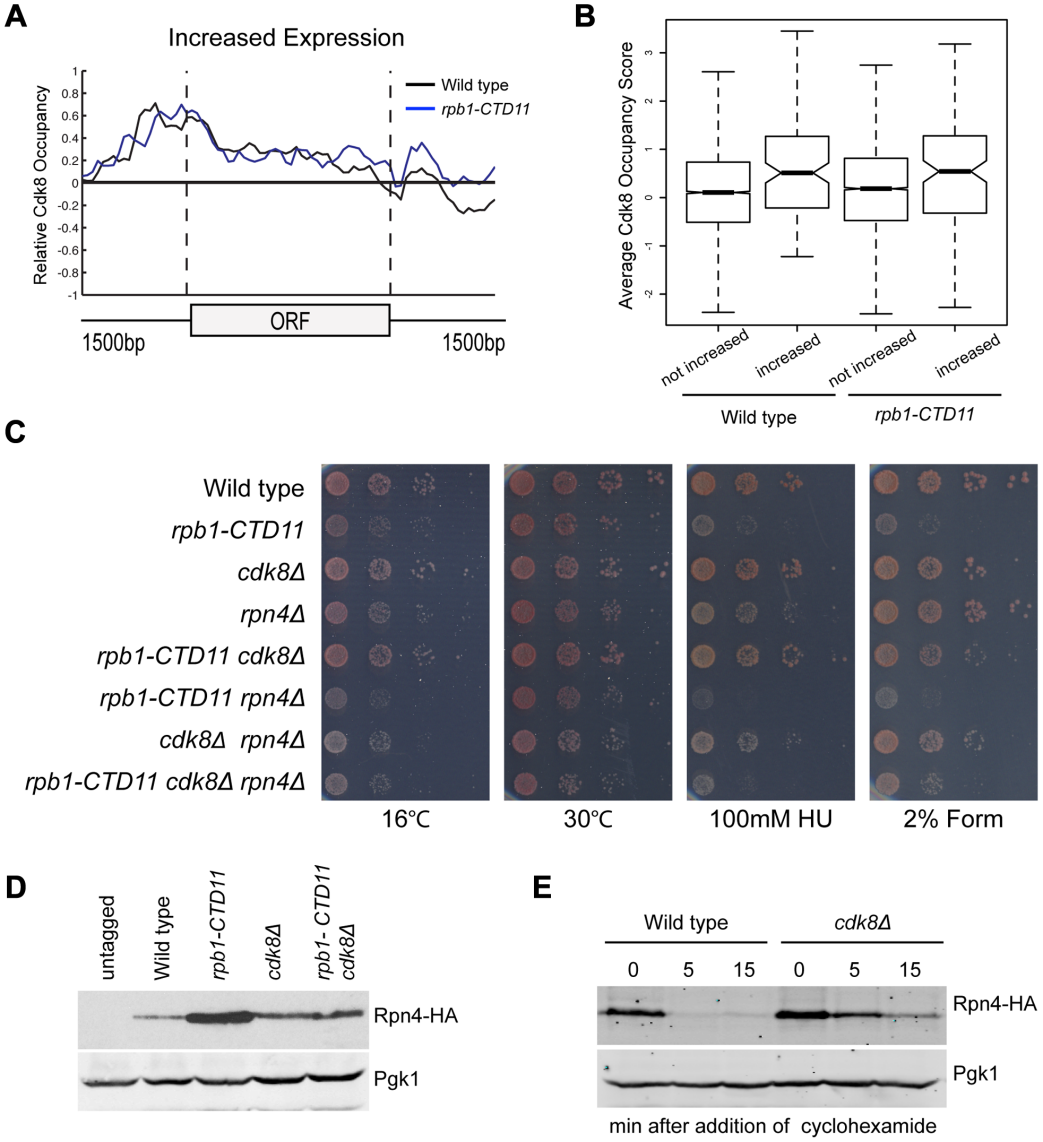
Regulation of Rpn4 levels partly mediated the suppression of *rpb1-CTD11* defects by loss of *CDK8*. (A) Cdk8 occupied the promoters of genes whose expression increased in the *rpb1-CTD11* mutant regardless of CTD length. (B) Boxplot comparing average Cdk8 occupancy scores at the promoters of genes whose expression increased in the *rpb1-CTD11* mutant (increased) to all other genes in the genome (not increased). Significantly higher Cdk8 occupancy occurred at the promoters of genes with increased expression levels in both the wild type and the *rpb1-CTD11* mutant. (C) The sensitivity of *rpb1-CTD11*, *cdk8Δ*, *rpn4Δ* single, double and triple mutants in the W303 background was tested by plating ten-fold serial dilutions on YPD media at 16, 30 and 37°C and YPD media containing the indicated concentrations of hydroxyurea or formamide. Deletion of *RPN4* abolished the suppression. (D) Immunoblot of Rpn4 protein levels identified an increase of Rpn4 in *rpb1-CTD11* mutants that was reduced upon deletion of *CDK8*. Pgk1 was used as a loading control. (E) Cdk8 regulated the stability of Rpn4 *in vivo*. Rpn4 protein stability was measured at the indicated time points under wild type and *cdk8Δ* conditions. Pgk1 was used as a loading control.

To understand the mechanism underlying the restoration of the transcription and RNAPII recruitment changes in the *rpb1-CTD11* mutant upon loss of *CDK8*, we first tried to understand the role of Cdk8 in regulating these genes. To determine if Cdk8 played a direct regulatory role at these genes, we generated a genome-wide map of Cdk8 occupancy under wild type conditions (Complete dataset can be found in array-express, code E-MTAB-1379). The average gene occupancy of Cdk8 showed clear enrichment at promoters, although we did identify Cdk8 binding to a small number of ORFs (Figure S5) [22], [23], [46]. Focusing on CTD-length dependent genes, we observed Cdk8 occupancy at the promoters of genes with increased mRNA levels in the *rpb1-CTD11* mutant (Figure 8A), while very little Cdk8 was observed at the set of genes with decreased levels (data not shown). Importantly, Cdk8 occupancy was not significantly altered in strains with a truncated CTD (Figure 8A). In both situations, the preferential association of Cdk8 with the genes having increased expression was significant even when compared to all genes in the genome (one-tailed, unpaired t-test p-value 0.0001079 for wild-type and 0.005898 for *rpb1-CTD11*, respectively), thus supporting a direct regulatory role for Cdk8 at these loci (Figure 8B). However, despite its significant association and robust effect on normalizing the expression levels of this set of genes, our gene expression analysis clearly showed that Cdk8 was not the sole regulator of these genes as these were generally normal in *cdk8Δ* mutants (Figure 6A) [47].

### The Suppression of Genes with Increased Levels in the *rpb1-CTD11* Mutant by Loss of *CDK8* Was through an Effect in Regulating the Levels of the Transcription Factor Rpn4

Using strict criteria, our profiles of *rpb1-CTD11* and *rpb1-CTD11 cdk8Δ* mutants revealed robust restoration of mRNA levels at 45% of the genes with increased expression levels in the *rpb1-CTD11* mutant and 24% of the genes with decreased levels when *CDK8* was deleted (Figure 6A). Among the genes with increased expression, those suppressed were involved in proteasome assembly and proteasome catabolic processes (Table S4). Consistently, these genes were primarily regulated by Rpn4 (Bonferroni corrected p value of hypergeometric test 1.06E-26). Of the genes with decreased expression, the suppressed set were mainly involved in iron transport, assimilation and homeostasis, however, no significantly associated transcription factors were identified.

Given that our data thus far suggested that the restoring effect was at the level of initiation and mediated by Cdk8, we concentrated our efforts in determining if Rpn4, the only transcription factor found to be significantly involved in regulating the expression of the suppressed set of genes, contributed to the suppression. First, we determined if *RPN4* was genetically required for the suppression of CTD truncation phenotypes by loss of *CDK8* by generating *rpb1-CTD11*, *cdk8Δ* and *rpn4Δ* single, double and triple mutants and testing their growth on different conditions. To test for specificity we also investigated whether the suppression was affected by *GCN4*, which encodes for a transcription factor involved in the regulation of the genes whose expression increased in the *rpb1-CTD11* mutant but not on those suppressed by deletion of *CDK8*. Deletion of *RPN4* in the *rpb1-CTD11 cdk8Δ* background abolished the suppression, indicating that *RPN4* was genetically required (Figure 8B; compare *rpb1-CTD11 cdk8Δ* to *rpb1-CTD11 cdk8Δ rpn4Δ*). In contrast, deletion of *GCN4* in the *rpb1-CTD11 cdk8Δ* background had no effect on the suppression, suggesting that the genetic interactions with *RPN4* were specific (Figure S8).

Considering that Rpn4 is a phospho-protein, we also tested the involvement of two previously identified phosphorylation sites that are important for its ubiquitin-dependent degradation [48]. Introduction of the *RPN4* S214/220A mutant restored the suppression in a *rpb1-CTD11 cdk8Δ rpn4Δ* strain in most of the conditions tested, thus demonstrating a general lack of involvement of these phosphorylation sites in the suppression (Figure S8 right panel: compare *rpb1-CTD11 cdk8Δ* and *rpb1-CTD11 cdk8Δ rpn4Δ*) [48]. Despite our inability to link Rpn4 phosphorylation to the suppression mechanism, the genetic analysis showed that the growth of *rpb1-CTD11 rpn4Δ* double mutants was more compromised than that of *rpb1-CTD11* mutants alone, indicating a clear dependence on Rpn4 function for maintaining *rpb1-CTD11* cell fitness (Figure 8B compare *rpb1-CTD11* and *rpb1-CTD11 rpn4Δ* mutants). This phenotypic pattern contrasted the apparent increase in Rpn4 function in a *rpb1-CTD11* mutant as suggested by our gene expression analysis, and indicated that mutating *CDK8* normalized, rather than abolished Rpn4 activity in *rpb1-CTD11* mutants. To test this hypothesis, we measured the levels of Rpn4 fused to a hemagglutinin (HA) tag in *rpb1-CTD11* and *cdk8Δ* single and double mutants. Consistent with an increase in Rpn4 function, Rpn4 protein levels were increased in *rpb1-CTD11* mutants compared to wild type cells (Figure 8D). Surprisingly, Rpn4 protein levels were reduced upon deletion of *CDK8* in the *rpb1-CTD11* mutant, consistent with the observed restoration in gene expression of Rpn4 target genes. In addition, the initial gene expression analysis as well as detailed RT-qPCR analysis of the *RPN4* locus did not detect significant alterations in *RPN4* mRNA levels in *rpb1-CTD11* and *CDK8* single and double mutants, suggesting that the effect of the CTD and Cdk8 on Rpn4 was most likely at the protein level (data not shown). In support of this and consistent with the slightly elevated level of Rpn4 in the *cdk8Δ* strain (Figure 8D), loss of *CDK8* increased the half-life of Rpn4 (Figure 8E). This suggested that Cdk8 was a regulator of Rpn4 stability *in vivo*.

## Discussion

Our genetic interaction, mRNA profiling, and RNAPII binding studies illuminated key linkages between CTD function, gene expression, mediator function, and the transcription factor Rpn4. We found distinct CTD- length dependent genetic interactions and gene expression alterations during steady state growth. The majority of the expression changes in the CTD mutants were in genes whose mRNA levels increased and these were accompanied by increased RNAPII binding across their coding regions. CTD truncation mutants were primarily defective in transcription initiation as suggested by our E-MAP profile of the *rpb1-CTD11* mutant and further supported by reporter assays. Removal of the mediator subunit, Cdk8, in cells with shortened CTD restored the original mRNA levels and RNAPII occupancy profiles at a subset of genes whose expression was increased in the CTD truncation mutant, highlighting an activating role for Cdk8 in gene expression regulation. In contrast, loss of *CDK8* also restored the reduced activation of the *INO1* gene exemplifying the more established repressive role for Cdk8. Finally and highly consistent with the expression results, shortening the CTD resulted in increased cellular amounts of the transcription factor Rpn4, which was normalized upon concomitant removal of *CDK8*. Underscoring its role, we found that *RPN4* was genetically required for the suppression of CTD truncation phenotypes by loss of *CDK8*.

The mRNA analysis identified genes whose expression levels during normal growth were dependent on CTD length, thus expanding the existing knowledge of CTD function *in vivo*, which has been derived from a primary focus on genes activated in response to specific conditions including *INO1* and *GAL10* [7]. Despite the CTD being essential for viability *in vivo*, we detected a seemingly low number of genes with altered expression levels in *rpb1-CTD11* mutants. We reconcile this with the fact that our shortest allele was four repeats above the minimum required for viability in *S. cerevisiae*, suggesting that we were predominantly assaying those genes most sensitive to changes in CTD length rather than the essential function of the CTD. Nonetheless, using stringent criteria our data identified a set of over 200 genes whose transcription was CTD length-dependent. As expected from the well-documented role of the CTD in transcription activation, about 40% of CTD-dependent genes had decreased expression. Surprisingly, we found that about 60% of CTD-dependent genes had increased expression. Functional analysis of the genes with increased or decreased expression upon CTD truncation revealed key differences in mRNA stability, transcriptional frequency, GO categories and associated transcription factors, suggesting differential effects on groups of genes with distinct properties. In addition, for both groups there was a high correlation between mRNA levels and RNAPII occupancy suggesting a direct effect on RNAPII function rather than changes in posttranscriptional RNA processing. Furthermore, truncating the CTD also caused changes in the association of Cet1 and H3K36me3 at genes whose expression was altered in the *rpb1-CTD11* mutant. Finally, our data linked the alterations observed at the genes with increased mRNA levels to changes in transcription initiation using promoter-fusion experiments. How this latter finding can be reconciled with the minor changes in TFIIB association at the promoters of these genes remains to be determined.

The increased mRNA levels and concurrent increase in occupancy of RNAPII in *rpb1-CTD11* mutants presents an interesting conundrum. Seemingly, these results pointed to a previously unreported inhibitory function of the CTD, as shortening it relieved the inhibition and resulted in higher RNAPII occupancy. However, we favor a model in which these relationships are reflective of a cellular stress response elicited by impairing CTD function. Consistent with this hypothesis, CTD truncation mutants displayed heightened sensitivity to a variety of stressors, as shown by others and us [4], [19], [49]. Furthermore, CTD truncation mutants had increased levels of Rpn4 protein and the genes that had increased mRNA levels tended to be regulated by Rpn4, consistent with their important contributions to the cellular stress response [50]–[52].

In addition, we investigated the molecular underpinnings of the well-established connection between Cdk8 and the RNAPII CTD. To this end, we found that deletion of *CDK8* normalized the expression of genes with increased mRNA levels in the CTD truncation alleles. This observation is consistent with the less-understood role for *CDK8* as an activator of transcription, likely acting by enhancing recruitment of RNAPII with a shortened CTD to its target genes. Given that Cdk8 was found to be preferentially associated with the promoters of these genes regardless of CTD length, it is likely that this represents a direct mechanism. Importantly, our data clearly showed that Cdk8 was not the sole regulator of this subset of genes as a single deletion of *CDK8* does not alter their expression. Thus, in wild type cells Cdk8 associated at these genes' promoters but it only enhanced transcription when CTD function was disrupted. This observations are in agreement with Cdk8's well-established role in the response to environmental signals [31], [53], [54]. Furthermore, we show that Cdk8's role in activating CTD-dependent genes with increased mRNA levels was in part mediated by increasing the protein levels of the transcription factor Rpn4, which we found to be genetically required for the suppression. Accordingly, the levels of Rpn4 protein correlated with the mRNA levels of Rpn4 targets genes in *rpb1-CTD11* and *cdk8Δ* single and double mutants. This is consistent with the known role of Cdk8 in regulating protein levels of transcription regulatory proteins and the established function of Rpn4 in activating gene expression as a result of stress [55]. Reminiscent of recent work by several groups showing that loss of Cdk8 stabilizes Gcn4 protein levels, our data on Rpn4 protein stability provided further support of a close linkage between Cdk8 and Rpn4, although the mechanistic details remain to be determined [56]–[58]. In addition, we note that not all suppressed genes are known targets of Rpn4, suggesting that it is likely not the only factor linking the RNAPII CTD and Cdk8 function.

The fact that removal of Cdk8 also suppressed defects in activated transcription suggested an entirely different relationship between the RNAPII-CTD and Cdk8 from the one described above, this time involving a negative role for Cdk8. This is exemplified by the *INO1* locus, where *rpb1-CTD11* mutants have decreased mRNA expression and RNAPII association when grown in inducing conditions, a defect that was restored upon deletion of *CDK8*. While reminiscent of the model postulating that Cdk8-catalyzed phosphorylation of the CTD prevents promoter binding of RNAPII and thus results in transcriptional repression, we do not think this is the mechanism of suppression described here [29]. First, deletion of *CDK8* had no alleviating effects on the bulk phosphorylation status of either full-length or truncated CTD. Second, deletion of *CDK8* alone under non-inducing conditions did not result in de-repression of *INO1*, in contrast to well-characterized Cdk8 target genes [47]. Lastly, despite our genome-wide Cdk8 occupancy data showing a reproducible, albeit slight, enrichment of Cdk8 at the *INO1* promoter, it does not meet our enrichment criteria, making it unclear if Cdk8 directly associates and functions at this locus (data not shown). In conclusion, our data revealed a tight link between Cdk8 and the RNAPII-CTD in transcription regulation, where Cdk8 can both enhance and repress transcription, the former in part mediated by regulating the levels of the transcription factor, Rpn4.

## Materials and Methods

### Yeast Strains, Plasmids and Growth Conditions

Strains and plasmids are listed in Supplementary materials. Partial, complete gene deletions or integration of a 3XFLAG tag was achieved via the one-step gene replacement method [59]. CTD truncations were created at the *RPB1* locus by addition of a TAG stop codon followed by a NAT resistance marker and confirmed by sequencing. As a control for E-MAP and gene expression analysis we used *RPB1-CTDWT*. This strain contained a NAT resistance marker following the endogenous stop codon. pRS314 [RPN4] and pRS314 [rpn4 S214/220A] were obtained from Dr. Youming Xie (Wayne State University School of Medicine). Reporter plasmids were generated by cloning 450 bp of the desired promoter into the Sal1 BamH1 sites of pLG669-Z [60].

### Epistasis Miniarray Profiling

E-MAP screens were performed and normalized as described previously [32]. Strains were screened in triplicate. Complete E-MAP profiles can be found in Supplementary Table S1.

### Microarrays Experiments and Analysis

Microarrays were performed in duplicate as previously described [61], [62]. Cultures were grown with a 24-well plate incubator/reader. Spiked in controls were used to determine global changes in mRNA levels. As no such changes were detected, the expression profiles were normalized to total mRNA levels, a more reproducible measure. Differentially expressed genes were determined by p value <0.01 and fold change >1.7 compared to wild type. Complete expression profiles can be found in Supplementary Table S2. Suppressed genes were determined as those having fold changes <1.1 in the *rpb1-CTD11 cdk8Δ* mutant. The Yeast Promoter Atlas database was used for transcription factor enrichment by performing a Hypergeometric test with Bonferroni correction (p value 0.05) [63]. “Biological Process” ontology annotated in the Bioconductor package org.Sc.sgd.db was used for GO enrichment using the conditional Hypergeometric test (adjusted p value <0.05) described in the following reference [64], [65]. Supplementary Table S3 and S4 contain a full list of significant GO terms.

### Chromatin Immunoprecipitation (ChIP)

Yeast cultures were grown in media containing 200 µM of inositol (uninduced) and switched to media lacking inositol for 4 hrs (induced) [45]. Cross-linking was done with 1% formaldehyde for 20 min. Chromatin was prepared as described previously [66]. 5 µl of anti-Rpb3 (Neoclone) was used. Crosslinking reversal and DNA purification were followed by qPCR analysis of the immunoprecipitated and input DNA. cDNA was analyzed using a Rotor-Gene 600 (Corbett Research) and PerfeCTa SYBR Green FastMix (Quanta Biosciences). Samples were analyzed from three independent DNA purifications and normalized to an intragenic region of Chromosome V [67]. Primers are listed in Supplementary materials.

### Genome-Wide ChIP-on-chip

ChIP-on-chip cultures were grown overnight in YPD, diluted to 0.15 OD600 and grown to 0.5–0.6OD600 units. Cross-linking and chromatin isolation were performed as above. 5 µl of anti-Rpb3 (Neoclone), 4.2 µl of anti-FLAG (Sigma) or 4 µl of anti-H3K36me3 (Abcam ab9050) were coupled to 60 µl of protein A magnetic beads (Invitrogen). DNA was amplified using a double T7 RNA polymerase method, labeled and hybridized as previously described [66]. Samples were normalized as described previously using the rMAT software [68]. Relative occupancy scores were calculated for all probes using a 300 bp sliding window. Rpb3 and H3K36me3 experiments were normalized to input while Flag-tagged factors were normalized to untagged controls. Samples were carried out in duplicate, quantile normalized and averaged data was used for calculating average enrichment scores. For ORFs, we averaged probes whose start sites fell within the ORF start and end positions, and for promoters we averaged probes mapping to 500 bp upstream of the ORFs. Enriched features had at least 50% of the probes contained in the feature above the threshold of 1.5. Enriched features were identified for each replicate and the overlap was reported as the significantly enriched set.

### ChIP-on-chip Visualization

CHROMATRA plots were generated as described previously [69]. In detail, relative occupancy scores for each transcript were binned into segments of 150 bp. Transcripts were sorted by their length and transcriptional frequency and aligned by their TSSs. Transcripts were grouped into five classes according to their transcriptional frequency as per Holstege *et al* 1998. Average gene profiles were generated by averaging all probes that mapped to genes of interest. For averaging, probes corresponding to ORFs were split into 40 bins while probes corresponding to UTRs were split into 20 bins.

### Reporter Assays

Reporter plasmids were transformed into wild type and *rpb1-CTD11* mutants and assayed as previously described [70]. Measurements were obtained from three independent cultures.

### Growth Assays

Overnight cultures grown on YPD or –TRP media were diluted to 0.5 OD600, 10-fold serially diluted and spotted onto YPD or –TRP plates with or without the indicated amounts of hydroxyurea (Sigma), formamide (Sigma), or on plates lacking inositol. Plates were incubated at the indicated temperatures for 2–4 days.

### Protein Blotting

Whole cell extracts were prepared from logarithmic growing cells by glass bead lysis in the presence of trichloroacetic acid. Immunoblotting was carried out with 3E10, 3E8, 4E12, 8WG16 (Millipore), YN-18 (Santa Cruz), Rpb3 (Neoclone), HA-Peroxidase (Roche) and Pgk1 (Molecular Probes) antibodies [43]. Immunoblots were scanned with the Odyssey Infrared Imaging System (Licor) or visualized with SuperSignal enhanced chemiluminescence (Pierce Chemical).

### Reverse Transcriptase PCR (RT-PCR)

RNA was extracted and purified using the Qiagen RNeasy Mini Kit. cDNA was generated using the Qiagen QuantiTect Reverse Transcription Kit. cDNA was analyzed by qPCR as described above. *INO1* mRNA levels were normalized to *ACT1* mRNA [7]. Samples were analyzed in triplicate from three independent RNA preparations.

### Protein Stability Assay

Overnight cultures were diluted to 0.3 OD600 and grown to 1.0 OD600. 10 OD600 units were collected to constitute time 0 and a final concentration of 100 ug/ml of cycloheximide (Sigma) was added to the remaining culture. 10 OD600 units were collected at the indicated time points. Proteins were extracted using trichloroacetic acid.

## Supporting Information

Figure S1 Sample genetic interaction network of CTD truncations mutants revealed CTD length-dependent genetic interactions. Subsets of genetic interaction profiles depicting genes involved in transcription and how they interacted with the CTD as it was progressively shortened. Blue and yellow represent aggravating and alleviating genetic interactions respectively. Gray boxes represent missing values.
(PDF)

Figure S2 Comparison of previously published Rpb3 genome-wide association profiles. (A) CHROMATRA plots of RNAPII occupancy [69]. Relative occupancy of previously published Rpb3 profiles across all transcripts sorted by their length and transcriptional frequency and aligned by their TSSs. Transcripts were grouped into five classes according to their transcriptional frequency as per Holstege *et al* 1998. (B) Chromosome plot of a 55-kilobase pair region on chromosome 5 (genomic positions 50,000–105,000).
(PDF)

Figure S3 Truncation of the RNAPII CTD leads to changes in the genome-wide association of transcription association factors. (A, B, C and D) CHROMATRA plots of relative occupancy of transcriptional associated factors [69]. Relative occupancy of TFIIB, Cet1, Elf1 and H3K36me3 across all transcripts sorted by their length and transcriptional frequency and aligned by their TSSs. Transcripts were grouped into five classes according to their transcriptional frequency as per Holstege *et al* 1998.
(PDF)

Figure S4 Deletion of *CDK8* suppressed CTD-associated growth phenotypes. (A) The sensitivity of CTD truncation mutants containing 11 or 12 repeats to known and novel growth conditions was suppressed by deleting *CDK8*. Ten-fold serial dilutions of strains containing the indicated CTD truncations with and without deletion of *CDK8* were plated and incubated on YPD media at 16, 30 and 37°C and YPD media containing the indicated concentrations of hydroxyurea or formamide. (B) Immunoblots of whole cell extracts with CTD phosphorylation specific antibodies. YN-18 detects the N-terminus of Rpb1 and was used as a control for Rpb1 protein levels. Rpb3 was used as a loading control.
(PDF)

Figure S5 Genome-wide Cdk8 occupancy plots agreed with previous reports. Average Cdk8 occupancy at all genes separated by transcriptional frequency revealed a preference of Cdk8 for binding to the promoter of highly transcribed genes (left) and confirmed that Cdk8 binding at coding regions was independent of transcriptional frequency (right).
(PDF)

Figure S6 *GCN4* was not involved in the suppression of *rpb1-CTD11* phenotypes by loss of *CDK8*. The sensitivity of *rpb1-CTD11*, *cdk8Δ* and *gcn4Δ* single, double and triple mutants in the W303 background was tested by plating ten-fold serial dilutions on YPD media at 16, 30 and 37°C and YPD media containing the indicated concentrations of hydroxyurea or formamide.
(PDF)

Figure S7 Phosphorylation of Rpn4 at S214/220 is not involved in the suppression of *rpb1-CTD11* defects by loss of *CDK8*. The sensitivity of *rpb1-CTD11*, *cdk8Δ*, *rpn4Δ* single, double and triple mutants carrying an empty vector, or a plasmid containing either *RPN4* or *RPN4 S214/220A* was tested by plating ten-fold serial dilutions on YPD media at 16, 30 and 37°C and YPD media containing the indicated concentrations of hydroxyurea or formamide.
(PDF)

Table S1 E-MAP profiles of *rpb1-CTD11, 12, 13, 20* and full length mutants.
(XLSX)

Table S2 Gene expression profile of strains containing 11 or 12 heptapeptide repeats with or without deletion of *CDK8* and strains containing 13 or 20 repeats or full length CTD (see attached excel file). M value is the log2 of the ratio between the two samples per gene.
(XLSX)

Table S3 Biological process gene ontology terms enriched in genes with increased or decreased mRNA levels in the *rpb1-CTD11* mutant.
(XLS)

Table S4 Biological Process gene ontology terms enriched in the subset of genes with increased or decreased mRNA levels that were suppressed by loss of *CDK8* in *rpb1-CTD11* mutants.
(XLS)

Table S5 Strains used in this study.
(XLS)

Table S6 Plasmids used in this study.
(XLS)

Table S7 Primers used in this study.
(XLS)
