# Supplementary figures and images for: High-Throughput Genetic and Gene Expression Analysis of the RNAPII-CTD Reveals Unexpected Connections to SRB10/CDK8
Source: PLoS Genet. 2013 Aug 29;9(8):e1003758. doi: 10.1371/journal.pgen.1003758 (PMC3757075; doi:10.1371/journal.pgen.1003758)

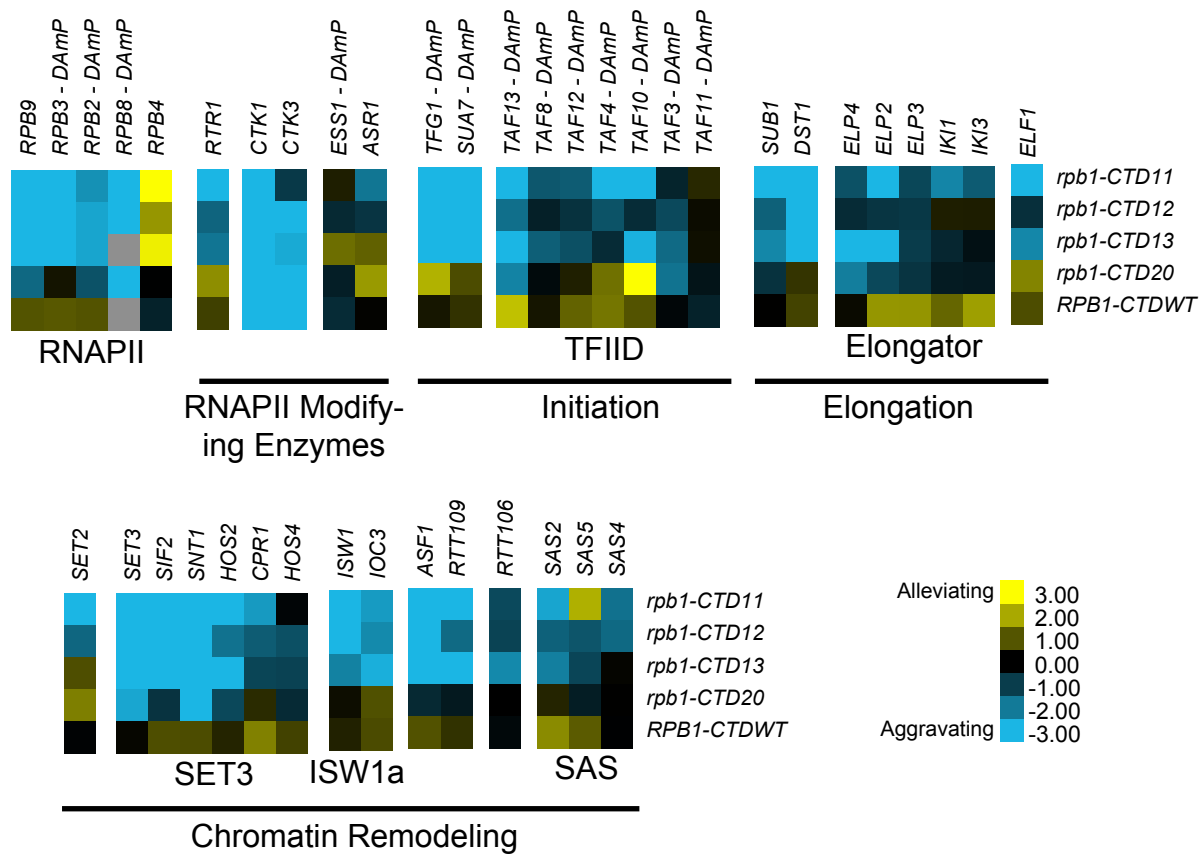

Supplement: Figure S1 — Sample genetic interaction network of CTD truncations mutants revealed CTD length-dependent genetic interactions. Subsets of genetic interaction profiles depicting genes involved in transcription and how they interacted with the CTD as it was progressively shortened. Blue and yellow represent aggravating and alleviating genetic interactions respectively. Gray boxes represent missing values. (PDF) [file pgen.1003758.s001.pdf]

**a**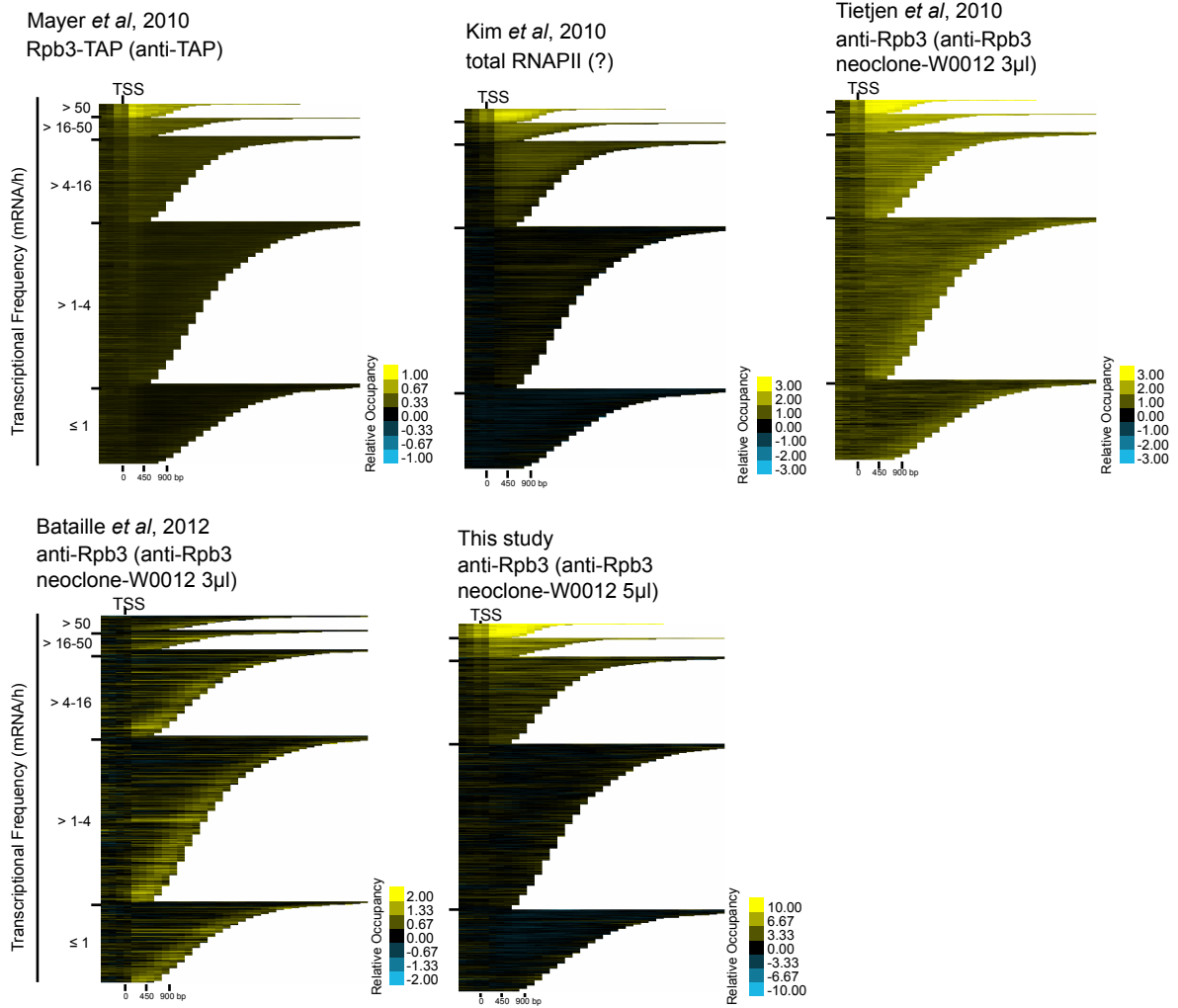**b**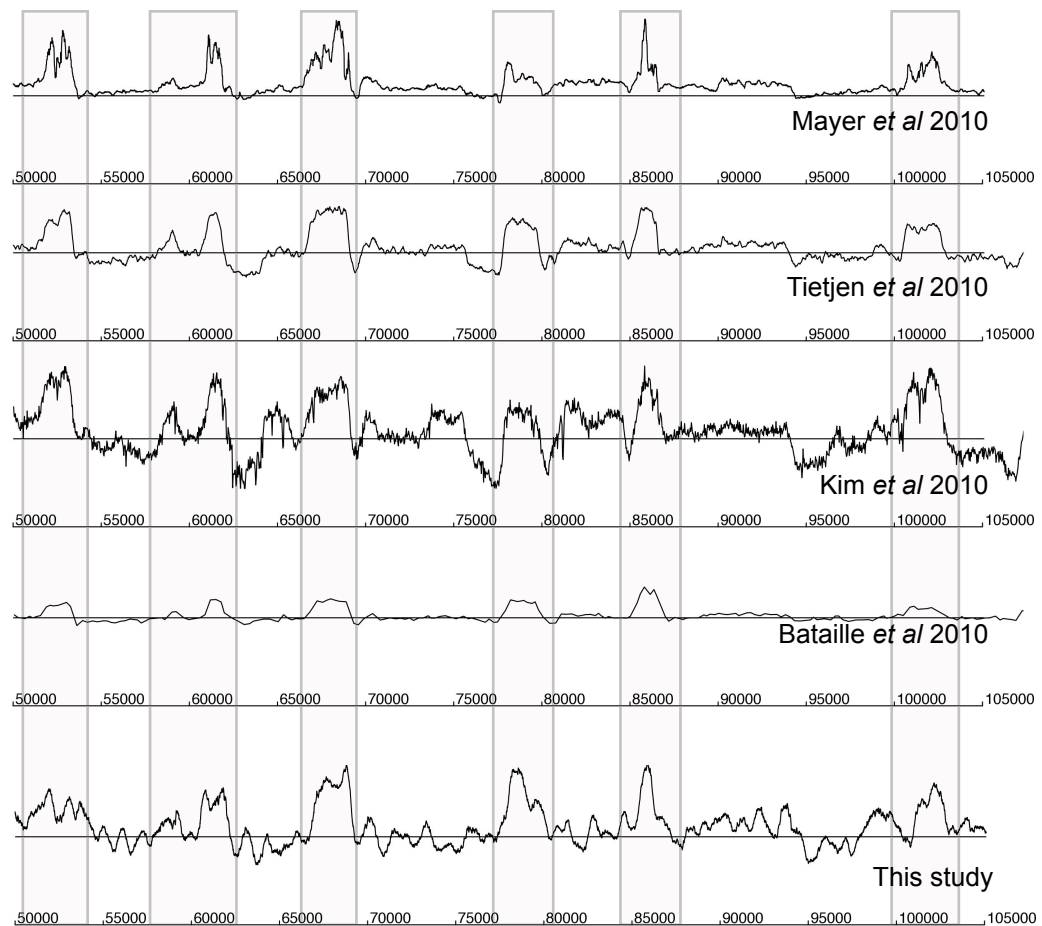

Supplement: Figure S2 — Comparison of previously published Rpb3 genome-wide association profiles. (A) CHROMATRA plots of RNAPII occupancy [69]. Relative occupancy of previously published Rpb3 profiles across all transcripts sorted by their length and transcriptional frequency and aligned by their TSSs. Transcripts were grouped into five classes according to their transcriptional frequency as per Holstege et al 1998. (B) Chromosome plot of a 55-kilobase pair region on chromosome 5 (genomic positions 50,000–105,000). (PDF) [file pgen.1003758.s002.pdf]

**a**

TFIIB-flag

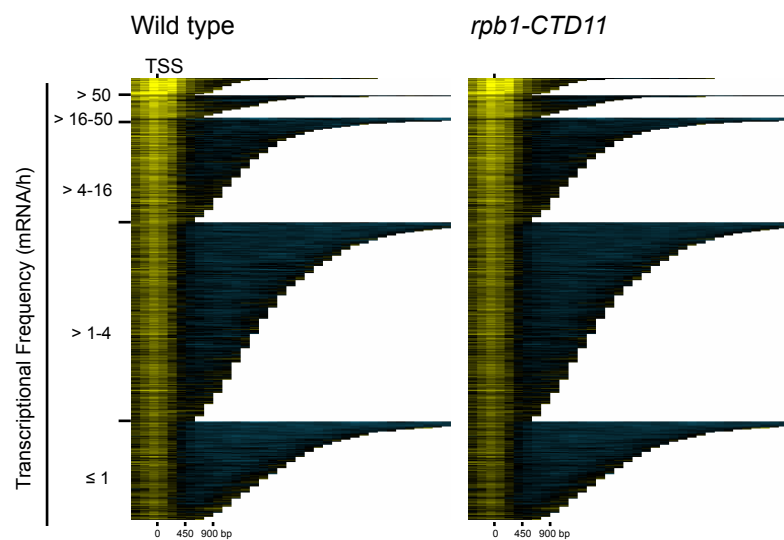**b**

Cet1-flag

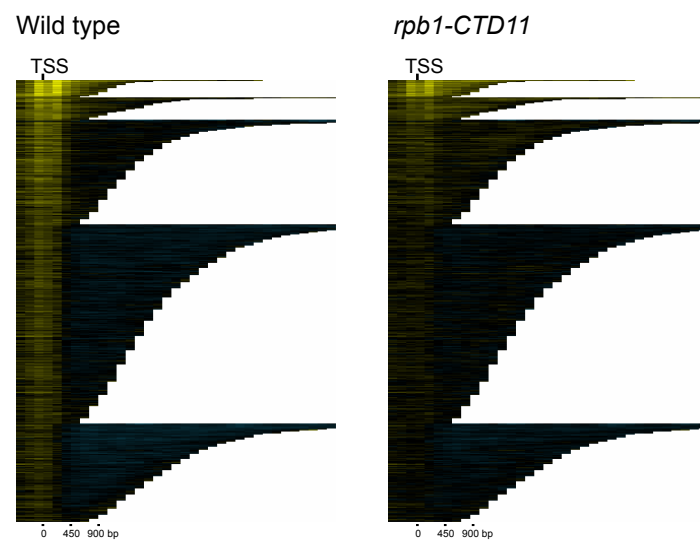**c**

Elf1-flag

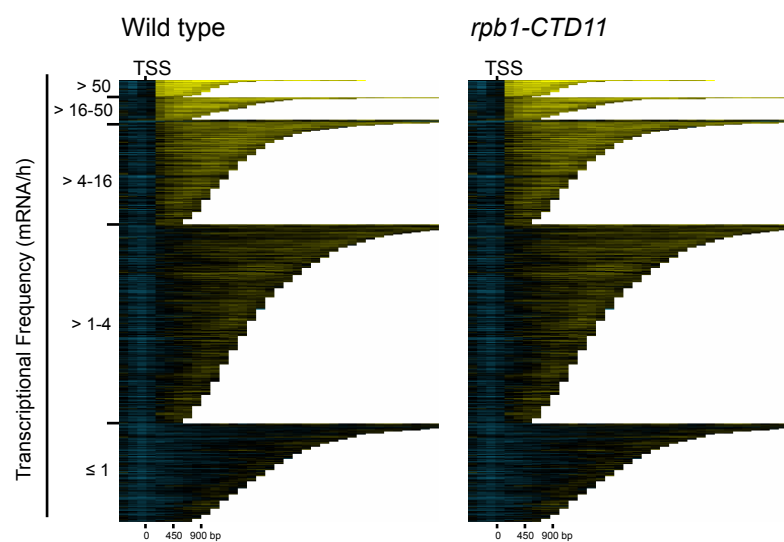**d**

H3K36me3

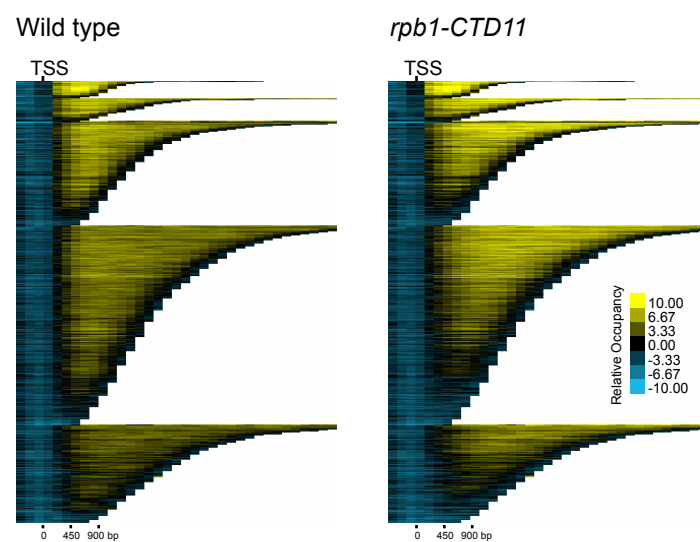

Supplement: Figure S3 — Truncation of the RNAPII CTD leads to changes in the genome-wide association of transcription association factors. (A, B, C and D) CHROMATRA plots of relative occupancy of transcriptional associated factors [69]. Relative occupancy of TFIIB, Cet1, Elf1 and H3K36me3 across all transcripts sorted by their length and transcriptional frequency and aligned by their TSSs. Transcripts were grouped into five classes according to their transcriptional frequency as per Holstege et al 1998. (PDF) [file pgen.1003758.s003.pdf]

a

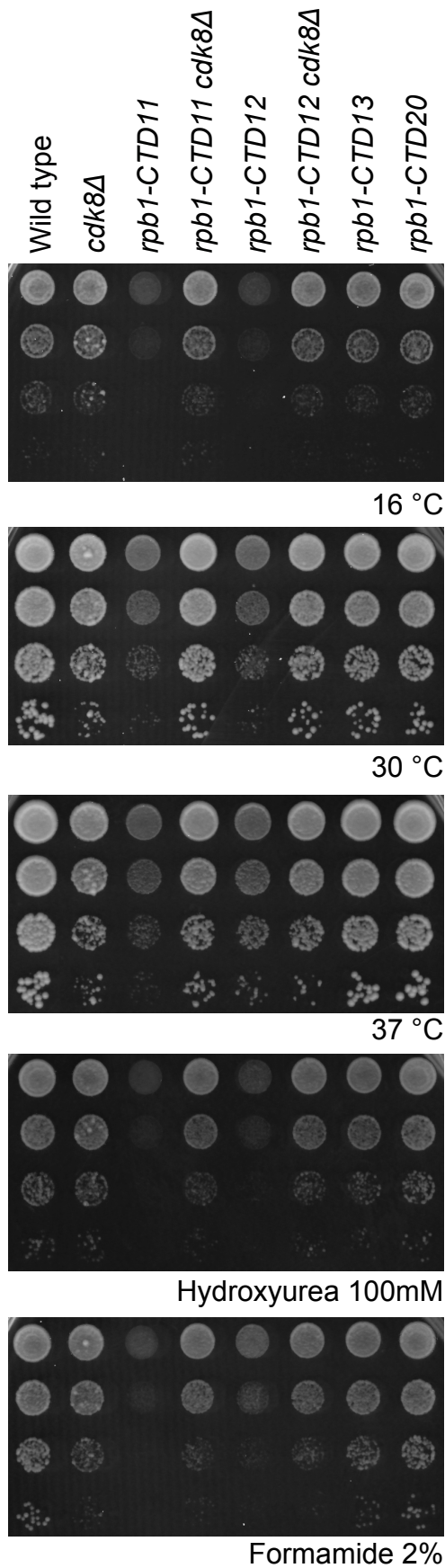

b

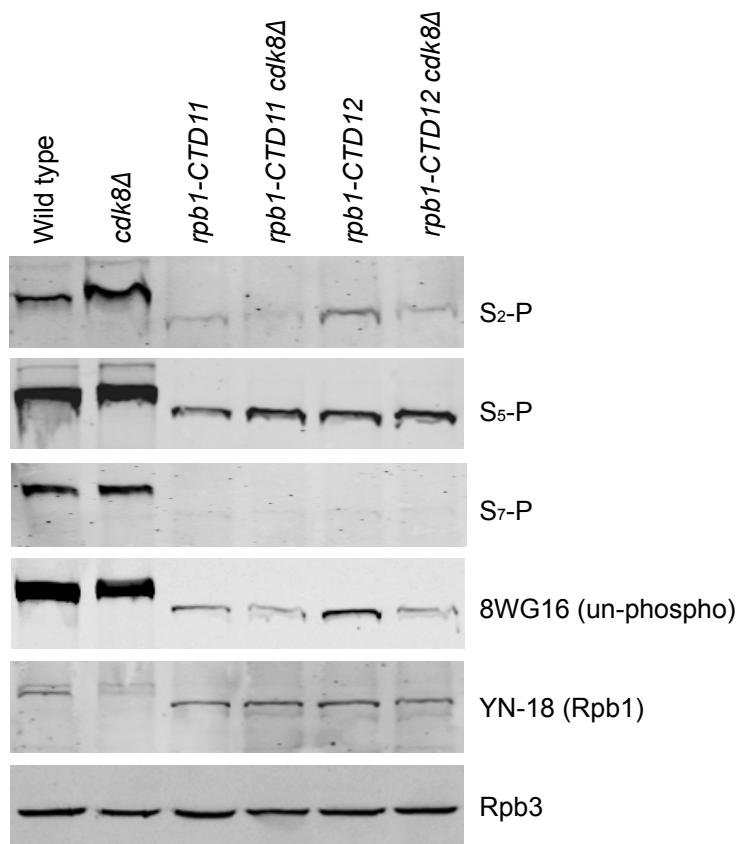

Supplement: Figure S4 — Deletion of CDK8 suppressed CTD-associated growth phenotypes. (A) The sensitivity of CTD truncation mutants containing 11 or 12 repeats to known and novel growth conditions was suppressed by deleting CDK8. Ten-fold serial dilutions of strains containing the indicated CTD truncations with and without deletion of CDK8 were plated and incubated on YPD media at 16, 30 and 37°C and YPD media containing the indicated concentrations of hydroxyurea or formamide. (B) Immunoblots of whole cell extracts with CTD phosphorylation specific antibodies. YN-18 detects the N-terminus of Rpb1 and was used as a control for Rpb1 protein levels. Rpb3 was used as a loading control. (PDF) [file pgen.1003758.s004.pdf]

All ORFs

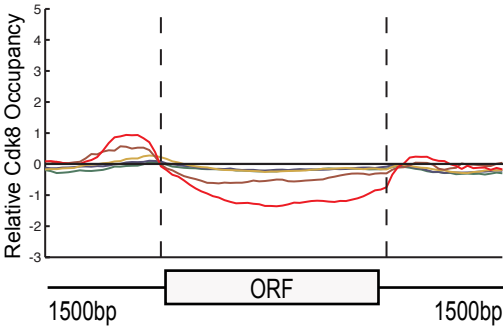

Enriched ORFs

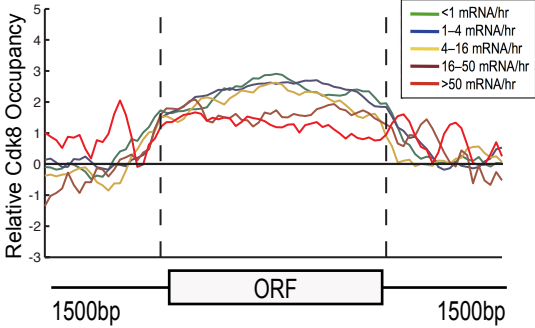

Supplement: Figure S5 — Genome-wide Cdk8 occupancy plots agreed with previous reports. Average Cdk8 occupancy at all genes separated by transcriptional frequency revealed a preference of Cdk8 for binding to the promoter of highly transcribed genes (left) and confirmed that Cdk8 binding at coding regions was independent of transcriptional frequency (right). (PDF) [file pgen.1003758.s005.pdf]

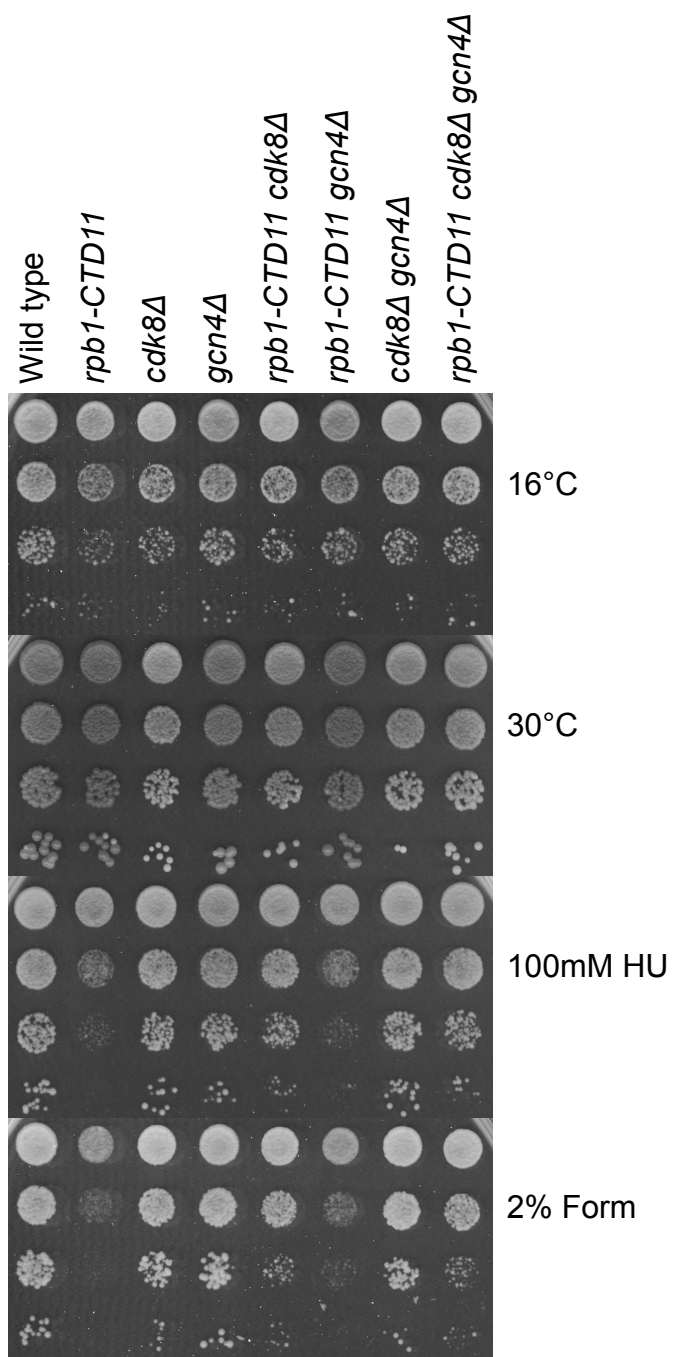

Supplement: Figure S6 — GCN4 was not involved in the suppression of rpb1-CTD11 phenotypes by loss of CDK8. The sensitivity of rpb1-CTD11, cdk8Δ and gcn4Δ single, double and triple mutants in the W303 background was tested by plating ten-fold serial dilutions on YPD media at 16, 30 and 37°C and YPD media containing the indicated concentrations of hydroxyurea or formamide. (PDF) [file pgen.1003758.s006.pdf]

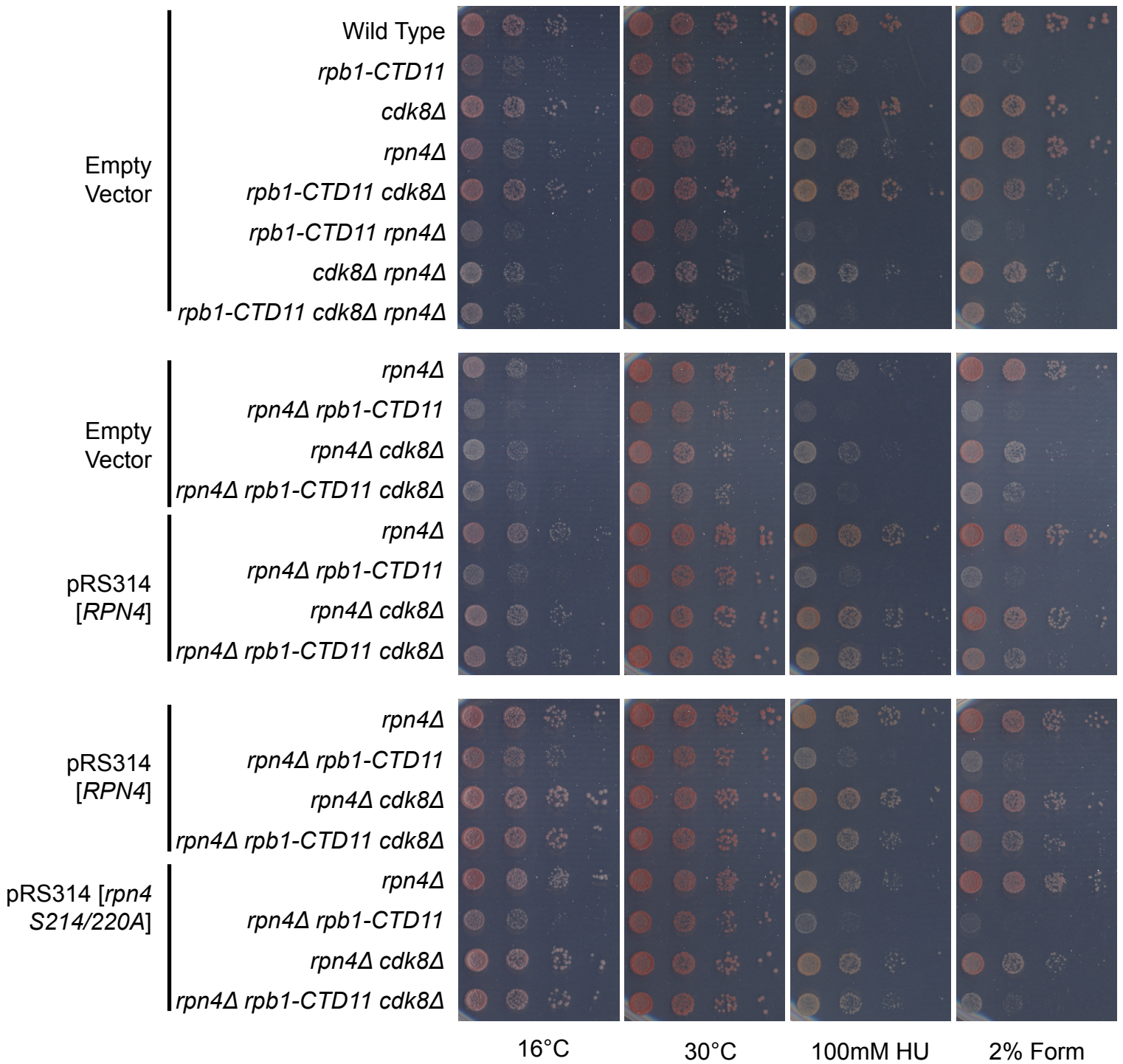

Supplement: Figure S7 — Phosphorylation of Rpn4 at S214/220 is not involved in the suppression of rpb1-CTD11 defects by loss of CDK8. The sensitivity of rpb1-CTD11, cdk8Δ, rpn4Δ single, double and triple mutants carrying an empty vector, or a plasmid containing either RPN4 or RPN4 S214/220A was tested by plating ten-fold serial dilutions on YPD media at 16, 30 and 37°C and YPD media containing the indicated concentrations of hydroxyurea or formamide. (PDF) [file pgen.1003758.s007.pdf]
